# Effect of Partner Violence in Adolescence and Young Adulthood on Blood Pressure and Incident Hypertension

**DOI:** 10.1371/journal.pone.0092204

**Published:** 2014-03-21

**Authors:** Cari Jo Clark, Susan A. Everson-Rose, Alvaro Alonso, Rachael A. Spencer, Sonya S. Brady, Michael D. Resnick, Iris W. Borowsky, John E. Connett, Robert F. Krueger, Shakira F. Suglia

**Affiliations:** 1 Department of Medicine, University of Minnesota, Minneapolis, Minnesota, United States of America; 2 Division of Epidemiology and Community Health, University of Minnesota, Minneapolis, Minnesota, United States of America; 3 Program in Health Disparities Research, University of Minnesota, Minneapolis, Minnesota, United States of America; 4 Independent Researcher, Atlanta, Georgia, United States of America; 5 Department of Pediatrics, University of Minnesota, Minneapolis, Minnesota, United States of America; 6 Division of Biostatistics, Biostatistical Design and Analysis Center, University of Minnesota, Minneapolis, Minnesota, United States of America; 7 Department of Psychology, University of Minnesota, Minneapolis, Minnesota, United States of America; 8 Department of Epidemiology, Columbia University, New York, New York, United States of America; Medical University Innsbruck, Austria

## Abstract

Intimate partner violence has adverse health consequences, but little is known about its association with hypertension. This study investigates sex differences in the relationship between intimate partner violence and blood pressure outcomes. Data included 9,699 participants from waves 3 (2001–02) and 4 (2008–09) of the National Longitudinal Study of Adolescent Health (51% female). Systolic (SBP) and diastolic (DBP) blood pressure and incident hypertension (SBP≥140 mmHg, DBP≥90 mmHg, or taking antihypertensive medication) were ascertained at wave 4. Intimate partner violence was measured at wave 3 with 8 items from the revised Conflict Tactics Scales. Separate victimization and perpetration scores were calculated. Sex-specific indicators of severe victimization and perpetration were created using the 66^th^ percentile among those exposed as a cut point. Sex-specific, linear and logistic regression models were developed adjusting for age, race, financial stress, and education. Thirty-three percent of men and 47% of women reported any intimate partner violence exposure; participants were categorized as having: no exposure, moderate victimization and / or perpetration only, severe victimization, severe perpetration, and severe victimization and perpetration. Men experiencing severe perpetration and victimization had a 2.66 mmHg (95% CI: 0.05, 5.28) higher SBP and a 59% increased odds (OR: 1.59, 95% CI: 1.07, 2.37) of incident hypertension compared to men not exposed to intimate partner violence. No other category of violence was associated with blood pressure outcomes in men. Intimate partner violence was not associated with blood pressure outcomes in women. Intimate partner violence may have long-term consequences for men's hemodynamic health. Screening men for victimization and perpetration may assist clinicians to identify individuals at increased risk of hypertension.

## Introduction

One in 10 US adolescents report exposure to physical intimate partner violence (IPV) in the prior year.[1] IPV is related to serious mental and physical health sequelae;[2] however, its relationship to hypertension, a condition affecting over one-third of the US adult population,[3] and a growing number of adolescents[4] is unclear. Psychosocial stress is a known risk factor for hypertension,[3] but little is known about stressors that occur over important developmental time periods such as the transition from adolescence to adulthood. Since high blood pressure has been shown to track from from adolescence into adulthod,[5] greater understanding of the potential early life psychosocial contributors to elevated blood pressure is warranted.

Prior research on the relationship between IPV and elevated blood pressure has focused on adult violence exposure and has found limited,[6] or no support for the association.[7]–[12] Existing research on the topic, however, is hindered by a predominance of cross-sectional designs and a lack of objectively measured blood pressure outcomes. Sex differences in the relationship between IPV and hypertension have been largely unexplored due to female-only samples which comprise a majority of the literature. The impact of being in a mutually violent relationship, a defining feature in up to half of violent relationships,[13]–[15] and the role of perpetration have not been examined. Therefore, more questions than answers remain as to whether IPV is related to blood pressure and hypertension status.

This study addresses these gaps by investigating sex differences in the relationship between exposure to IPV victimization and perpetration in adolescence and young adulthood (between average ages 16 and 22) and blood pressure outcomes (systolic blood pressure (SBP), diastolic blood pressure (DBP), incident hypertension) in adulthood (average age 29).

## Materials and Methods

### Sample

The study sample included participants of Waves 3 (2001–2002) and 4 (2008–2009) of the National Longitudinal Study of Adolescent Health (Add Health). Add Health is a longitudinal study of a nationally representative sample of adolescents in grades 7–12 in the U.S. during the 1994–95 school year.[16] Of the 12,288 participants who participated in baseline and waves 3 and 4, 10,058 reported a relationship at the Wave 3 interview in which IPV status was ascertained and of these 9,699 were selected for this analysis because they had non-missing values on covariates. For the incident hypertension analyses, the sample size was restricted to participants without a self-reported health provider diagnosis of high blood pressure or hypertension at Wave 3 (N = 9,157).

### Ethics Statement

Participants provided written informed consent. The original study was approved by the institutional review board of the University of North Carolina, Chapel Hill.

### Measures

#### Blood Pressure and Hypertension

Blood pressure was measured at Wave 4. A detailed description of the methods involved, including cuff size, arm position and observer training has been published elsewhere.[17] Briefly, three readings at 30-second intervals were taken after a 5-minute seated rest using a factory calibrated, Microlife BP3MC1-PC-IB oscillometric blood pressure monitor; the last two readings were averaged and used in analyses. Three percent (n = 296) of participants reported using antihypertensive medication per a medication review;[18] therefore, their underlying blood pressure is unknown. It is common to remove such observations from analyses. However, doing so has been shown to introduce negative bias and reduce power.[19] Adding a constant that represents a realistic estimate of the treatment effect has been shown to substantially mitigate these limitations.[19] Therefore, a constant was added to the treated blood pressure measurements representing the average efficacy of a standard dose of antihypertensive medication (9 mmHg for SBP and 6 mmHg for DBP).[20] Hypertension was defined by SBP≥140 mmHg, DBP≥90 mmHg, or taking antihypertensive medication.

#### Intimate Partner Violence

Frequency of physical and sexual IPV and IPV-related injury was assessed at the Wave 3 interview using 4 items measuring victimization (threatened by partner with violence, pushed or shoved, or had something thrown at you that could hurt; partner slapped, hit or kicked you; partner made you have sexual relations when you did not want to; you had an injury, such as a sprain, bruise, or cut because of a fight with your partner) and 4 parallel items measuring the participant's perpetration of IPV based on the Revised Conflict Tactics Scales.[21] IPV was assessed in relationships that had occurred between summer 1995 (average participant age 16 years) and the Wave 3 interview (average participant age 22 years). Relationships chosen for assessment involved sexual intercourse, were current relationships of individuals selected for a couple's sub-sample, or were considered “important ” which was defined as those being longer term, recent, or involved marriage, co-habitation, or resulted in a pregnancy.

Rasch modeling was used to construct continuous scales of violence victimization and perpetration by modeling the conditional probabilities of responding yes to each item given its severity and the true but unobserved violence exposure level of each person. Items that are less frequently reported are treated as being more severe than those more frequently reported. The nature of physical violence supports this treatment since acts of low/moderate violence severity are the most common forms of IPV among dating and married couples in the U.S.[22], [23] The model is generalized to account for whether the event occurred in more than one relationship and more than once in a relationship.[24] Incorporating all available information creates a more parsimonious model that better discriminates between adolescents with low and high violence exposure and produces more adequate spread of items along the linear measurement summary scale. Rasch modeling has been used to create scales of violence exposure among children, and for war related trauma and IPV.[24]–[26]

The victimization and perpetration scores were combined into one variable with the following categories: no exposure; moderate victimization and/or perpetration; severe victimization but not severe perpetration; severe perpetration but not severe victimization; and both severe victimization and perpetration. Since there are no defined cut points for severe IPV using Rasch modeling, severe IPV was delineated by the 66^th^ percentile of the score among those reporting any exposure determined separately for men and women. Additional cut points were also examined to ascertain whether study findings were dependent upon the cut point.

#### Other Covariates

Sociodemographic variables included sex, self-reported race/ethnicity (categorized by the authors as Hispanic, Non-Hispanic white, black and other) and Wave 3 measures of age, educational attainment, and financial distress, defined by an affirmative response to any of 7 situations in which the respondent's household was unable to pay for various household and medical expenses.

### Statistical Analysis

Study variables were tabulated by exposure to IPV and sex. Chi-square tests were used to examine sex differences in IPV victimization and perpetration. Multivariable linear regression was used to test the relationship between IPV reported at Wave 3 and blood pressure assessed at Wave 4 adjusting for confounders (age, race-ethnicity, education, financial distress). Analyses were performed separately by sex given documented differences in women's and men's exposure to IPV[27], [28] and sex differences in emotional, behavioral, and physiologic responses to stress.[23], [28] Logistic regression was used to test the relationship between IPV and incident hypertension among participants who had not reported a health provider diagnosis of high blood pressure or hypertension at Wave 3. All models were repeated using alternate cut points for severe IPV (50^th^ and 80^th^ percentiles). Design effects and unequal probability of selection were accounted for according to Add Health user guidance.[29]

## Results

Mean age at baseline (Wave 3) was 21.83 (95% CI: 21.60, 22.06) years. Blood pressure measurement occurred approximately 7.01 (95% CI: 6.99, 7.03) years later (Wave 4). Participants were 50.56% female (N = 5388), 69.70% non-Hispanic white (N = 5626), 14.73% non-Hispanic black (n = 1932), 4.09% non-Hispanic other (n = 690), and 11.48% Hispanic (n = 1451). Thirty-three percent (n = 1430) of males and 46.90% (n = 2527) females reported IPV exposure. Women were more likely than men to report any victimization (women 34.42%, n = 1872; men 29.47%, n = 1227, Chi-square p-value = <.01) and any perpetration (women 37.96%, n = 2094; men 20.83%, n = 896, Chi-square p-value = <.01).

Tables 1 and 2 provide participant characteristics by sex and exposure to IPV. Compared to whites, blacks and Hispanics were more likely to be in the severe perpetration category and blacks and Hispanic women were more likely to have experienced both severe victimization and perpetration than no IPV exposure. Those who reported no violence exposure also reported less financial distress and greater educational attainment than those exposed to IPV. Compared to men exposed to IPV, men not exposed to IPV had lower mean SBP and DBP. Mean SBP and DBP did not significantly differ across levels of IPV exposure for women.

**Table 1:** Participant Characteristics by Sex and Level of Intimate Partner Violence Exposure, Men (N = 4,311).

	Total (N = 4311)	None (N = 2881)	Moderate Victimization and/or Perpetration (N = 845)	Severe Victimization^a^ (N = 265)	Severe Perpetration^a^ (N = 131)	Severe Victimization + Severe Perpetration^a^ (N = 189)
Age, yrs, mean (95% CI)	21.94 (21.69, 22.18)	21.88 (21.63, 22.14)	22.08 (21.80, 22.37)	21.76 (21.34, 22.18)	22.21 (21.75, 22.68)	22.18 (21.79, 22.57)
Race/ethnicity, n (%)						
Non-Hispanic White	2537 (69.94)	1751 (71.76)	474 (68.91)	165 (71.09)	60 (57.61)	87 (54.67)
Non-Hispanic Black	769 (14.19)	477 (13.05)	167 (15.14)	42 (14.78)	27 (13.01)	56 (26.21)
Non-Hispanic Other	326 (4.09)	211 (3.82)	65 (3.91)	24 (6.10)	13 (4.82)	13 (5.41)
Hispanic	679 (11.78)	442 (11.37)	139 (12.03)	34 (8.03)	31 (24.56)	33 (13.71)
Education, yrs, mean (95% CI)	13.00 (12.81, 13.19)	13.18 (12.99, 13.38)	12.64 (12.42, 12.85)	12.89 (12.50, 13.29)	12.80 (12.16, 13.44)	12.08 (11.61, 12.56)
Financial Distress, n (%)	1292 (29.95)	733 (25.40)	298 (35.56)	120 (43.71)	56 (39.28)	85 (46.88)
SBP, mean (95% CI)	129.94 (129.32, 130.55)	129.58 (128.87, 130.28)	130.32 (129.00, 131.64)	130.12 (128.10, 132.14)	130.66 (128.18, 133.13)	132.74 (130.00, 135.48)
DBP, mean (95% CI)	81.86 (81.35, 82.37)	81.50 (80.95, 82.04)	82.44 (81.45, 83.42)	82.14 (80.24, 84.03)	82.84 (81.04, 84.65)	83.53 (81.16, 85.90)

**Note**: ^a^severe IPV defined by the 66th percentile; SBP = systolic blood pressure; DBP = diastolic blood pressure.

**Table 2:** Participant Characteristics by Sex and Level of Intimate Partner Violence Exposure, Women (N = 5,388).

	Total (N = 5388)	None (N = 2861)	Moderate Victimization and/or Perpetration (N = 1507)	Severe Victimization^a^ (N = 279)	Severe Perpetration^a^ (N = 361)	Severe Victimization + Severe Perpetration^a^ (N = 380)
Age, yrs, mean (95% CI)	21.72 (21.49, 21.96)	21.80 (21.56, 22.04)	21.67 (21.41, 21.93)	21.78 (21.41, 22.14)	21.43 (21.12, 21.75)	21.59 (21.19, 21.99)
Race/ethnicity, n (%)						
Non-Hispanic White	3089 (69.45)	1754 (72.95)	815 (67.37)	182 (72.72)	160 (57.87)	178 (58.91)
Non-Hispanic Black	1163 (15.27)	541 (13.38)	358 (16.55)	32 (8.80)	112 (22.71)	120 (22.72)
Non-Hispanic Other	364 (4.10)	187 (4.04)	102 (3.72)	18 (5.53)	32 (5.38)	25 (3.82)
Hispanic	772 (11.18)	379 (9.63)	232 (12.35)	47 (12.95)	57 (14.04)	57 (14.55)
Education, yrs, mean (95% CI)	13.24 (13.06, 13.43)	13.49 (13.28, 13.70)	13.17 (12.94, 13.39)	12.66 (12.31, 13.02)	12.79 (12.49, 13.10)	12.47 (12.18, 12.75)
Financial Distress, n (%)	1921 (36.66)	811 (28.64)	614 (41.67)	133 (49.06)	167 (55.67)	196 (51.94)
SBP, mean (95% CI)	120.34 (119.79, 120.89)	120.07 (119.35, 120.78)	120.77 (119.78, 121.76)	120.97 (118.90, 123.03)	120.45 (118.49, 122.42)	120.20 (118.57, 121.82)
DBP, mean (95% CI)	77.18 (76.76, 77.61)	77.05 (76.49, 77.62)	77.08 (76.39, 77.77)	78.69 (77.06, 80.31)	77.65 (76.06, 79.24)	77.02 (75.69, 78.35)

Notes: * p-value<.05. a severe IPV defined by the 66th percentile.

**Figure 1:**
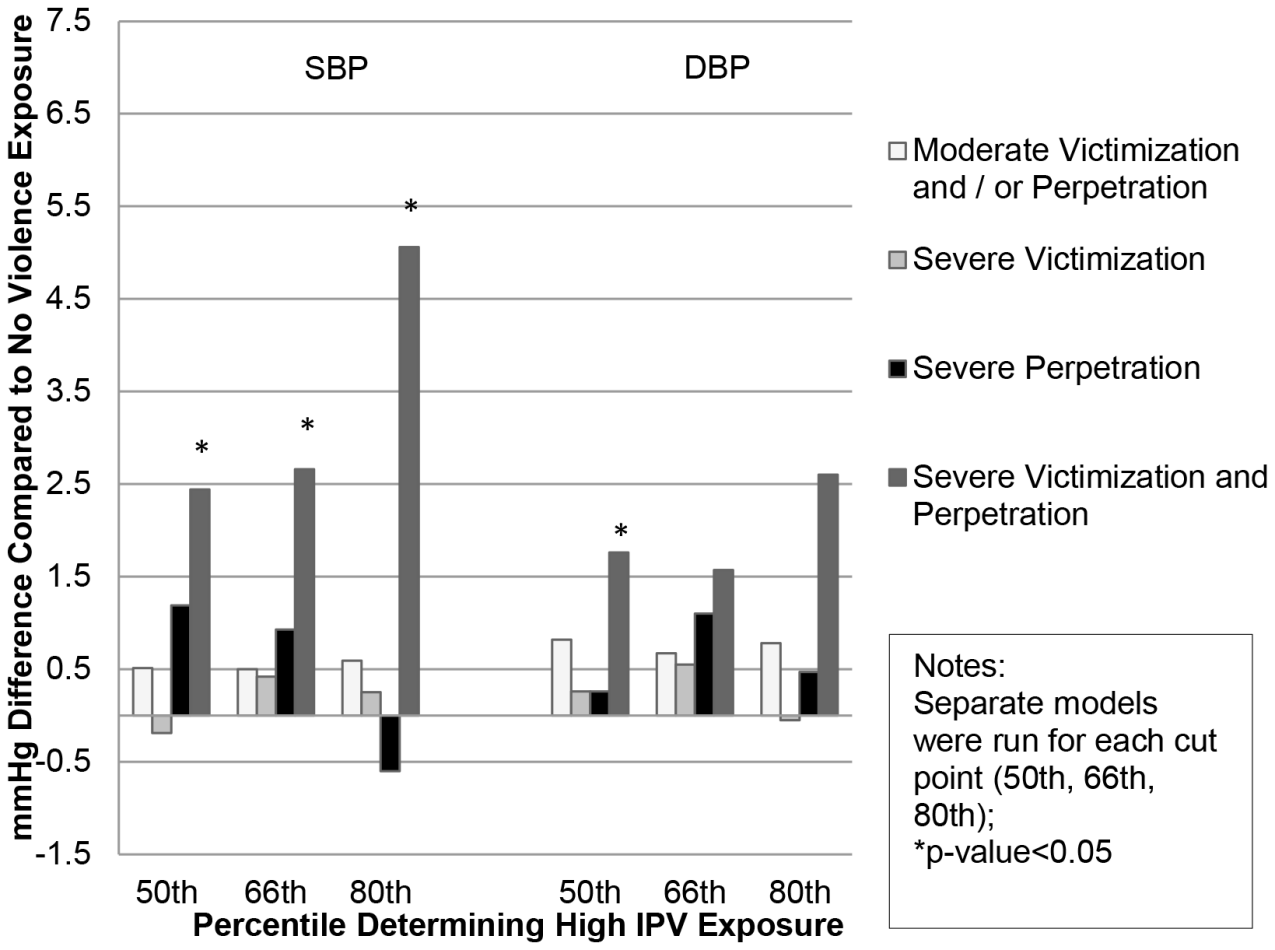
Difference in Blood Pressure by IPV Type and Cut Point, Men (N = 4311).

**Figure 2:**
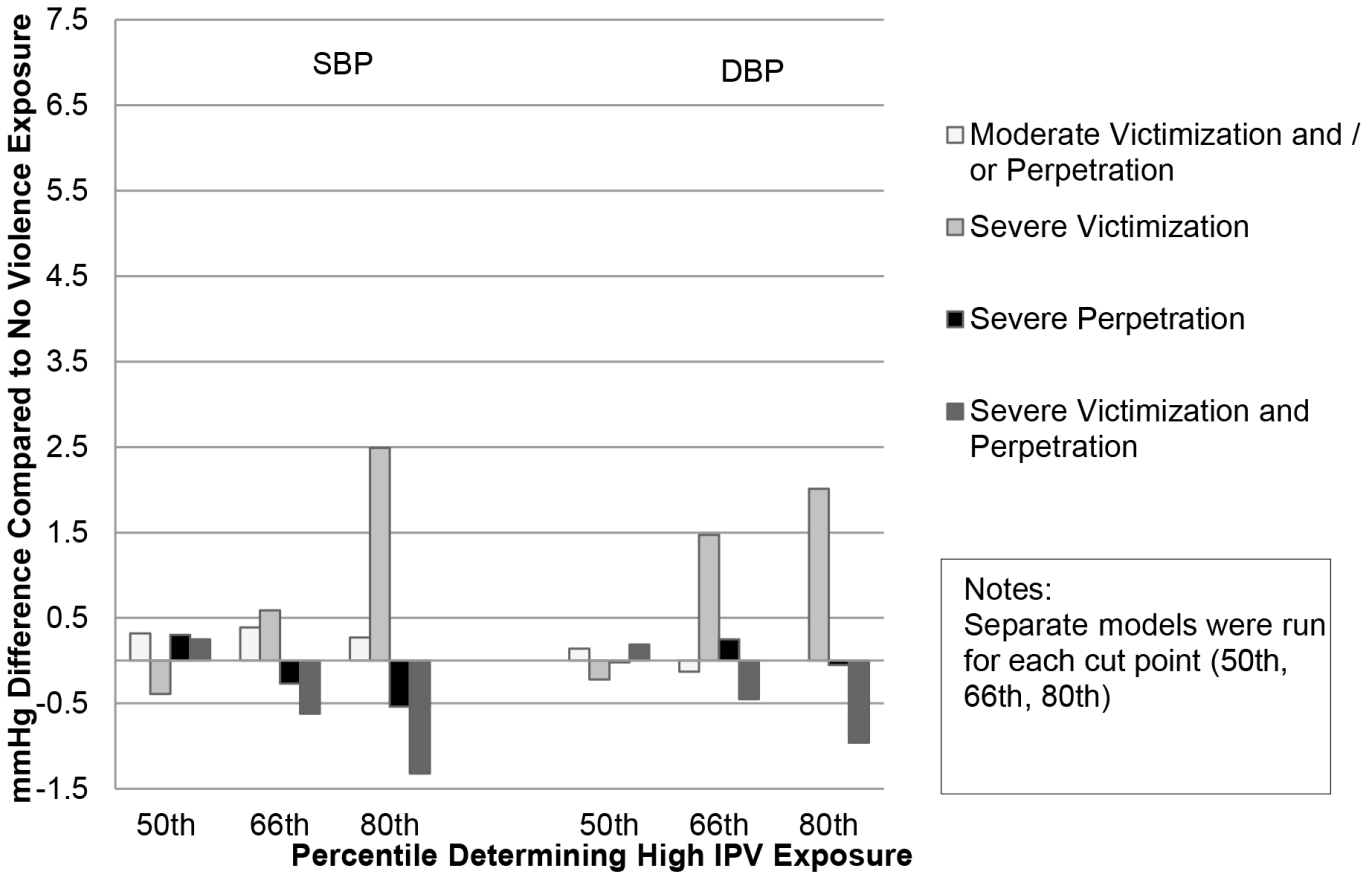
Difference in Blood Pressure by IPV Type and Cut Point, Women (N = 5338).

In models adjusting for confounders (Table 3), men experiencing both severe victimization and severe perpetration had a 2.66 mmHg (95% CI: .05, 5.28) higher SBP compared to those with no IPV exposure. Findings were similar for DBP although the magnitude of the effect was smaller and not statistically significant. The other categories of IPV were not statistically associated with blood pressure in men and IPV was not associated with blood pressure in women. Findings were robust to choice of severity cut point (Figures 1 & 2).

**Table 3:** Relationship Between Intimate Partner Violence and Systolic and Diastolic Blood Pressure by Sex (N = 9,699).

	Systolic Blood Pressure		Diastolic Blood Pressure	
	Men (N = 4311)	Women (N = 5388)	Men (N = 4311)	Women (N = 5388)
	Estimate (95% CI)	Estimate (95% CI)	Estimate (95% CI)	Estimate (95% CI)
No Violence Exposure	Ref	Ref	ref	ref
Moderate Victimization and / or Perpetration	.50 (−.89, 1.88)	.39 (−.78, 1.56)	.67 (−.30, 1.64)	−.13 (−.99, .73)
Severe^ a^ Victimization	.42 (−1.85, 2.68)	.59 (−1.51, 2.68)	.55 (−1.47, 2.56)	1.47 (−.28, 3.21)
Severe^ a^ Perpetration	.93 (−1.67, 3.54)	−.27 (−2.28, 1.74)	1.10 (−.85, 3.06)	.25 (−1.43, 1.93)
Severe^ a^ Victimization and Perpetration	2.66 (.05, 5.28)*	−.62 (−2.36, 1.12)	1.57 (−.74, 3.87)	−.45 (−1.85, .95)

Notes: * p-value<.05. ^a^severe IPV defined by the 66th percentile.

Among 9,157 participants not reporting hypertension in Wave 3, 1,671 (19.32%) had hypertension at Wave 4 (males: 26.48%, N = 1072; females: 12.21%, N = 599) (Table 4). In adjusted models, men who had experienced both severe victimization and perpetration had a 59% higher odds (OR = 1.59, 95% CI: 1.07, 2.37) of hypertension in adulthood compared to those who had not experienced IPV. The other categories of IPV were not statistically associated with blood pressure or incident hypertension in men and IPV was not associated with incident hypertension in women. The increased odds of hypertension among men exposed to both victimization and perpetration was robust to the choice of severity cut point.

**Table 4:** Relationship between Intimate Partner Violence Exposure and Incident Hypertension by Sex (N = 9,157).

	**MEN (N = 4090)**		
	50^th^	66th	80^th^
	OR (95% CI)	OR (95% CI)	OR (95% CI)
No Violence Exposure	Ref	Ref	Ref
Moderate Victimization and / or Perpetration	1.17 (.86, 1.59)	1.09 (.83, 1.42)	1.15 (.90, 1.47)
Severe Victimization	1.08 (.75, 1.56)	1.35 (.90, 2.02)	1.17 (.68, 2.02)
Severe Perpetration	1.10 (.69, 1.76)	1.39 (.80, 2.39)	1.79 (.99, 3.22)
Severe Victimization and Perpetration	1.61 (1.15, 2.25)*	1.59 (1.07, 2.37)*	1.62 (.91, 2.89)
	**WOMEN (N = 5067)**		
	50^th^	66th	80^th^
	OR (95% CI)	OR (95% CI)	OR (95% CI)
No Violence Exposure	Ref	Ref	Ref
Moderate Victimization and / or Perpetration	1.23 (.86, 1.77)	1.19 (.89, 1.60)	1.20 (.90, 1.59)
Severe Victimization	1.10 (.70, 1.71)	1.30 (.80, 2.13)	1.37 (.74, 2.54)
Severe Perpetration	1.01 (.66, 1.53)	1.10 (.68, 1.79)	1.08 (.68, 1.71)
Severe Victimization and Perpetration	1.23 (.83, 1.83)	.97 (.60, 1.56)	.80 (.42, 1.53)

Notes: * p-value<.05.

## Discussion

This study found that joint exposure to both severe victimization and severe perpetration during adolescence or young adulthood is associated with higher blood pressure and incident hypertension in men. These findings make a unique contribution to the literature on the physical health impact of IPV and are strengthened by the use of data from a large, nationally representative sample, objectively assessed blood pressure outcomes, and assessment of IPV prior to the measurement of blood pressure. Additional strengths include the examination of sex differences and joint modeling of IPV severity and directionality.

In this study, women were more likely than men to report victimization and perpetration. The finding that more women than men report IPV victimization is similar to criminal justice statistics[30] and numerous large nationally-representative studies,[15], [23], [31] including prior research using Add Health data.[13], [32] The finding that more women than men report perpetrating IPV is consistent with a meta-analysis on sex differences in perpetration,[27] other studies using nationally representative data,[15] and prior Add Health research.[13] Gendered differences in reporting may account for this finding. In prior research, men have been shown to underreport their perpetration of IPV,[28] potentially leading to misclassification.

In the present study, men had higher blood pressures and higher rates of incident hypertension compared to women which is consistent with existing literature.[3] However, 19% of the sample had hypertension, which is higher than other nationally representative samples of young adults ages 25–32 including NHANES (4.60%).[33] Prior Add Health research on this topic suggests that masked hypertension, measurement techniques, and sample composition might account for the differences.[33]

Findings regarding women are consistent with prior research demonstrating no association between IPV and hypertension [7]–[12] but are contrary to what was expected. Women generally report greater distress[23] and are more likely to smoke[34] and drink alcohol[35] in response to violence – plausible mediators of the hypothesized violence – blood pressure relationship. While not significant, study findings did suggest that when a very high cut point is used to determine severity, that blood pressure might be elevated among women who have been severely victimized. This finding was not statistically significant potentially due to low power given the small number of women reporting this level of exposure. IPV related changes in blood pressure have been noted in an older cohort of women (ages 25–42).[6] Older women are at greater risk of high blood pressure than younger women[3] and may have had longer-term exposure to IPV. Hemodynamic impacts of IPV may be detectable only among older women. The measurement of IPV used in this study might also have hampered this exploration. While the study did incorporate IPV items that disproportionately impact women, such as sexual violence and injuries, it did not measure emotional abuse.The only prior study to find a relationship between IPV and incident hypertension did so with a measure of current severe emotional abuse; no relationship was detected for lifetime physical or sexual IPV.[6] The present study did not distinguish between current and past abuse and none of the prior studies investigated the relationship between IPV and SBP or DBP limiting the ability to compare findings. On balance, study findings do not provide strong support for a relationship between IPV and blood pressure outcomes in young women. Further research that includes emotional abuse and that followed women into midlife could clarify this relationship.

In the present study, the most robust associations were among men. The only prior examination of sex differences found no relationship between IPV victimization and self-reported diagnosis of high BP in men[35] similar to the findings of the present study. However, among men exposed to both severe victimization and perpetration, blood pressure levels and the odds of incident hypertension were consistently higher than in those without IPV exposure. The more consistent findings among men might be explained by prior research on acute stress that found greater blood pressure responses in males than females.[36], [37] However, prior research on marital stress has shown the opposite,[38] or no sex effect.[39], [40] Prior research has not simultaneously modeled violence victimization and perpetration and has examined sex differences in blood pressure in cohorts older than the Add Health participants. Since men are more likely than women to develop high blood pressure before the age of 45,[3] the impact of IPV may be detectable only among men in this young cohort. Sex differences in violence reporting, combined with a high prevalence of relationships in which there is both victimization and perpetration suggest that research is needed that accounts for nuanced differences in men's and women's experiences of violence and how they relate to blood pressure outcomes. This study is a step in that direction, but clearly additional research is needed.

The study's findings are tempered by several factors. Residual confounding is possible and potential mediators such as smoking, excessive alcohol usage, and body mass index were not examined. Further research is needed to examine potential pathways linking severe victimization and perpetration in men and blood pressure related outcomes. IPV was self-reported since most violent incidents are not disclosed to verifiable sources such as service providers or law enforcement.[31], [41], [42] The present study used items from subscales of the CTS to measure physical and sexual IPV as well as IPV related injury, but did not assess severe emotional violence, which in prior research was associated with incident hypertension among a sample of women.[6] The items also do not measure the context of violence which could impact the individual's emotional and physiologic reaction. The limited number of items used to measure IPV precludes the creation of separate scores for physical and sexual violence. Since physical and sexual IPV frequently co-occur the study team chose to jointly measure these forms of violence to maximize the amount of data used to determine IPV exposure status. However, heterogeneity in the impact of violence by type cannot be ruled out. This study did not distinguish heterosexual from bi- or homosexual relationship history, which is a noteworthy focus of future research. Finally, blood pressure was only measured at Wave 4 so blood pressure change from Wave 3 cannot be examined and the incident hypertension outcome used in this manuscript is based on measurements at a single point in time. A diagnosis of hypertension requires elevated blood pressure levels during at least 2 clinic visits,[43] highlighting the need for clinical research on this topic.

## Conclusions

This study provides evidence that exposure to IPV is linked to elevated blood pressure and incident hypertension in men. While only severe IPV was investigated in this study, the effect sizes range from 2 to 5 mmHg suggesting public health relevance since average reductions of as little as 2 mmHg on a population basis can meaningfully reduce cardiovascular disease and all-cause mortality.[44] Including men in IPV screening efforts and assessing both victimization and perpetration may provide a more nuanced understanding of individuals at increased risk of hypertension.
